# A Longitudinal Evaluation of Bone Mineral Density Across a Macrocycle in Highly Trained Female Athletes: A Systematic Review

**DOI:** 10.3390/sports14040162

**Published:** 2026-04-17

**Authors:** Georgia M. Black, Madison Wells, Brooke L. Devlin

**Affiliations:** School of Human Movement and Nutrition Sciences, Faculty of Health, Medicine and Behavioural Sciences, St Lucia Campus, University of Queensland, Brisbane 4072, Australiab.devlin@uq.edu.au (B.L.D.)

**Keywords:** bone mineral density, female, athlete, bone adaptation, low energy availability

## Abstract

Bone health in highly trained female athletes is critical for performance and long-term wellbeing, yet systematic evidence regarding seasonal changes remains limited. The main objective of this systematic review (PROSPERO ID: 420251230393) is to determine changes in bone mineral density (BMD) and bone mineral content (BMC) across the sport macrocycle in highly trained female athletes, encompassing both elite and collegiate (NCAA) populations. Six databases were searched for studies published between 2010 and 2025, with inclusion requiring female athletes, BMD/BMC measurements, and longitudinal assessment across a macrocycle. Fourteen studies involving 522 premenopausal athletes were included, with dual-energy X-ray absorptiometry measurements conducted approximately six months apart. Study quality was assessed using the NIH Quality Assessment Tool for Observational Cohort and Cross-Sectional Studies and indicated a predominantly good quality. Five studies reported no significant change in BMD/BMC, five demonstrated improvements, three reported mixed findings across sports or athlete subgroups, and one reported a significant decline. Only two studies attempted to account for all three primary confounders—menstrual cycle status, dietary intake, and physical activity monitoring—while seven reported no confounding variables. While bone health appears largely maintained across the sport macrocycle in highly trained premenopausal female athletes, these findings should be interpreted cautiously given the inadequate confounder reporting, heterogeneous sport exposures, variability in skeletal sites measured, and inconsistent measurement timing. Future research must comprehensively assess these variables alongside sport-specific skeletal measurements to identify athletes at risk of bone health deterioration.

## 1. Introduction

Bone mineral density (BMD)—the mineral content per volume of bone, measured using dual-energy X-ray absorptiometry (DXA) [1]—is a critical determinant of skeletal strength and fracture risk, and is influenced by a combination of genetic, hormonal, and mechanical factors [2]. Peak bone mass is typically achieved in early adulthood, after which BMD naturally declines with ageing [3]. These declines underpin the disproportionate burden of osteoporosis and osteopenia in women, with osteoporosis being up to four times more prevalent in females than males [4]. Physical activity—particularly weight-bearing and high-impact exercise—is among the most powerful modifiable determinants of BMD, and female athletes consistently demonstrate higher BMD at key skeletal sites compared to non-athletic populations [5,6]. This osteogenic advantage is most pronounced in sports involving high mechanical loading such as gymnastics, weightlifting, and basketball, while athletes in non-weight-bearing disciplines such as swimming and cycling show comparatively attenuated bone adaptations [7,8]. Notably, the skeletal benefits of sport participation may persist beyond athletic careers, potentially delaying age-related bone loss [1,9]. The variation in mechanical loading across off-season, preseason, and competition phases of the sport macrocycle may therefore differentially influence skeletal adaptation, providing the mechanistic rationale for examining seasonal changes in bone health in athletic populations [10,11].

Despite this general advantage, approximately 18–34% of female athletic populations still report low BMD [12,13], with low-BMD athletes 4.5 times more likely to develop a stress fracture [14]. This prevalence may in fact be underestimated, as current BMD reference ranges are derived from non-athletic populations. Given that athletes possess inherently elevated BMD from training, an athlete who has sustained meaningful bone loss may still fall within population-normal limits on standard DXA assessment, masking genuine skeletal compromise and delaying clinical intervention [15]. This risk is closely linked to the Female Athlete Triad, first described by the ACSM in 1992 as the interrelationship between disordered eating, amenorrhea, and osteoporosis [16] and later broadened to Relative Energy Deficiency in Sport (RED-S) to reflect its applicability to male athletes and wider health consequences [17]. Central to both frameworks is low energy availability (LEA), defined as dietary intake minus exercise energy expenditure falling below 30 kcal/kg of fat-free mass per day [17], which is associated with poor skeletal and menstrual health and, if chronic, may have irreversible consequences for long-term bone health in female athletes [17].

Training loads, dietary intake, and hormonal status are well-established modulators of bone health in athletes [18], yet their interrelatedness across a macrocycle remains poorly understood. Fluctuations in training volume and intensity across periodised phases may inadvertently induce periods of LEA [19,20], which suppress the hypothalamic–pituitary–ovarian axis, disrupt menstrual function, and reduce circulating oestrogen—removing its protective effect on bone [21]. Concurrently, energy deficiency independently suppresses insulin-like growth factor-1 (IGF-1), and the combined presence of both deficiencies further exacerbates bone metabolism alterations [22,23]. Despite these well-characterised mechanisms, longitudinal evidence examining how these factors collectively evolve across a macrocycle and impact BMD remains limited. For the purposes of this review, “seasonal changes” refers to longitudinal within-athlete changes in BMD and BMC across one or more macrocycle phases, reflecting the heterogeneity of measurement designs across included studies.

Understanding how these interrelated factors collectively influence bone health across a macrocycle is therefore of applied relevance for female athletes, coaches, and clinicians. This systematic review aims to synthesise existing evidence on seasonal changes in bone health among premenopausal female athletes, with a focus on both impact and non-impact sports. Given the equivocal findings in the current literature, this review seeks to clarify the expected patterns of change in bone health over time and critically evaluate the quality of research, particularly in relation to how well studies account for known confounding factors affecting bone health.

## 2. Materials and Methods

A systematic review was registered in the International Prospective Register of Systematic Reviews (PROSPERO ID: 420251230393) and conducted using PRISMA (Preferred Reporting Items for Systematic Reviews and Meta-Analyses, 2020) guidelines (Supplementary Materials S2) [24]. Table 1 demonstrates the PICO (exposure in replacement of intervention) inclusion criteria for studies in this systematic review. For the purposes of this review, highly trained athletes were defined as those competing at collegiate level or above, consistent with the athlete classification framework previously proposed [25].

Electronic systematic literature searches were performed via a computer in the PubMed, Embase, Scopus, Central (Cochrane), Cinahl, and SportDISCUS databases from 2010 to 2 April 2025. The search was restricted to studies published from 2010 onwards to capture evidence of contemporary relevance to elite women’s sport [26] and the emergence of the RED-S framework [27], which has shaped how bone health in female athletes has been conceptualised and investigated. The databases were searched for title and abstract for the terms [“female athlete” OR “sportswoman” OR “female” OR “woman”] AND [“athlete” OR “sports”] AND [“bone health” OR “bone mineral density” OR “bone mineral content”] AND [“season*” OR “competiti*”]. Search strategies were adjusted for each database by using the Systematic Review Accelerator (SRA) tool [24]. Studies that were deemed eligible were uploaded into EndNote 20 (Clarivate Analytics, Philadelphia, PA, USA) by database. Once compiled, the papers were uploaded into Covidence (https://www.covidence.org, Melbourne, Australia accessed on 1 May 2025) [28] to be de-duplicated and screened, firstly by title and abstract and finally by full-text review.

Two researchers independently screened studies based on title and abstract according to the inclusion/exclusion criteria. Studies were excluded if they did not meet the PICO criteria outlined in Table 1 (e.g., non-female athletes, absence of BMD/BMC outcomes, or no longitudinal seasonal comparison). Then, the same two researchers (GB, MW) reviewed the selected full texts. Discrepancies were resolved by consensus. If no consensus could be reached, a third reviewer was available for a final decision, but this was not required. Reference lists of selected articles were also screened to identify additional relevant studies not captured by the electronic search. Grey literature sources, including Google Scholar and the Australian Institute of Sport (AIS) research repository, were also searched using search terms consistent with the electronic database strategy; no additional eligible studies were identified through this process.

**Figure 1:**
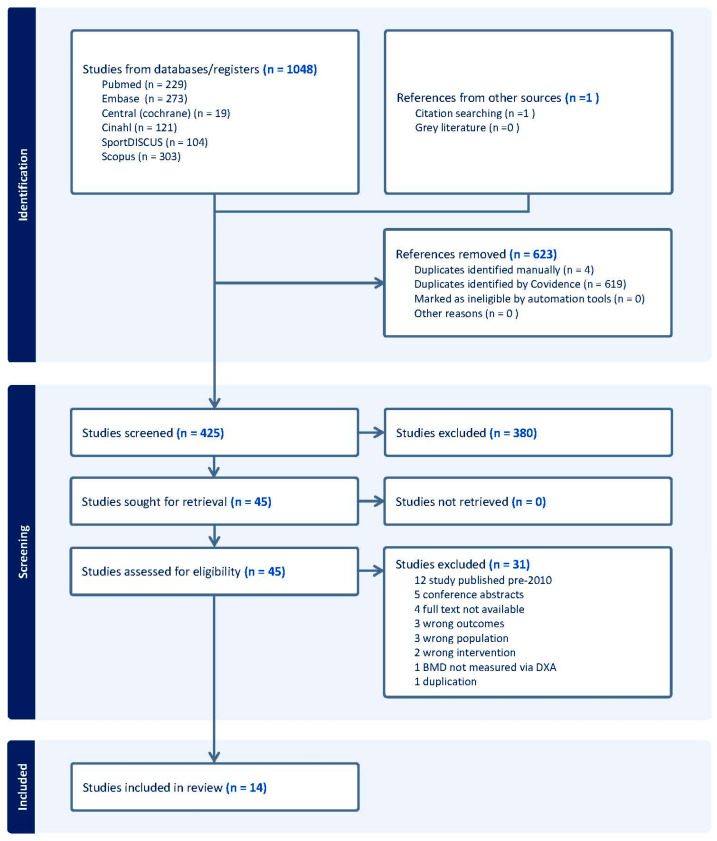
PRISMA diagram of study selection.

The search produced 1048 results (229 PubMed, 273 Embase, 303 Scopus, 18 Central (Cochrane Library), 121 Cinahl, and 104 SportDISCUS); the software removed 623 duplicates. The remaining 425 articles were screened for eligibility based on title and abstract by two researchers. This process removed a further 380 articles. The remaining articles were determined for final eligibility based on the inclusion and exclusion criteria listed in Table 1. Of the 44 articles selected for full-text review, 31 studies were excluded. Additionally, screening of the reference lists of included articles identified one further article that was included in the review, with 14 studies included in the review. The PRISMA flow diagram depicted in Figure 1 further describes the inclusion and exclusion process used to determine the final number of studies included in the review.

One researcher (MW) extracted the following data: author and year of publication; studied population (number, sex, age, sport); skeletal site; time between measurements; DXA changes and whether there was an increase, maintenance or reduction in BMD or bone mineral content (BMC); and dietary, training load and menstrual cycle reporting. All extracted data were subsequently cross-checked by a second researcher (GB) to verify accuracy and completeness. To provide a synthesis of the results from included studies, a descriptive analysis was performed. A quantitative synthesis was considered but deemed not feasible given the substantial heterogeneity across included studies in sport disciplines, skeletal sites assessed, measurement timepoints, and the inconsistent reporting of effect sizes and comparable statistical metrics across studies.

Two researchers independently assessed the methodological quality of included studies using the NIH Quality Assessment Tool for Observational Cohort and Cross-Sectional Studies [27]. Of the 14 questions listed, the “outcome assessors blinded” question was removed due to its lack of relevance to observational research data. Each paper was reviewed independently by two authors (GB, BD) to determine its quality as “good” (≥11/13 questions answered yes, >85%), “fair” (9–10 questions answered yes, 70–85%), or “poor” (<70%). 

## 3. Results

The flowchart of study selection is presented in Figure 1. Following the screening process, 14 studies were included in the review. Table 2 demonstrates individual study characteristics. A total of 522 female athletes with an average age of 21.4 ± 1.8 were included in this review. Eight studies used a cohort comprising NCAA division I or II athletes [29,30,31,32,33,34,35,36] and four studies investigated elite team sport athletes [37,38,39,40]. One study [41] investigated Swedish athletes across both national and international competition levels, and one studied Canadian Olympic athletes [42]. Studies were characterised based on the impact loading of their respective sports [43], with repetitive low-impact endurance sports classified separately from repetitive non-impact sports due to their distinct cyclical ground-contact loading profile. Where studies report data from multiple sports [34,36,41], findings are presented separately by sport in Table 2 to facilitate sport-specific comparison; however, each study is counted once in the total study count.

Two studies exclusively assessed bone changes in the repetitive non-impact sport of rowing [33,42]. One study investigated cross country athletes [35], two studies exclusively assessed high-impact sports [29,39], and five studies assessed odd-impact sports including rugby sevens [38], rugby union [37], soccer [31], lacrosse [30] and European handball [40]. The remaining studies assessed populations across varied impact-level sports within the same study [32,34,36,41]. Of the three primary confounding variables—training load, dietary intake, and menstrual cycle status—only two studies attempted to account for all three [30,35]. Only one study reported directly quantified training load [42], while two studies assessed physical activity as a proxy using questionnaire or accelerometry-based methods [30,35]. Four studies included information regarding athlete’s menstrual cycle status [29,31,33,42]; three studies reported only dietary intake [30,32,42]. Seven studies did not report on any confounding variable [34,36,37,38,39,40,41].

Across high-impact sports, the majority of studies reported either unchanged or improved BMD and/or BMC across the competitive macrocycle. Four [34,36,39,41] of the six included studies demonstrated significant improvements at one or more skeletal sites, while the remaining two reported no significant change [29,32]. Findings in odd-impact sports were mixed. Reported outcomes included increases in BMC with unchanged BMD [30,38], concurrent improvements in both BMD and BMC in one study [34], and increases in BMC without assessment of BMD [40]. The remaining studies reported no significant changes in BMD or BMC across a macrocycle [31,32,36,37]. In repetitive low-impact endurance sports, one study reported significant reductions in BMD across multiple skeletal sites over a 12-month period [35], while another demonstrated unchanged total BMD alongside site-specific improvements [41]. In repetitive non-impact sports, findings were variable. Two studies reported significant improvements in BMD with unchanged BMC [36,42] and one study reported concurrent increases in both BMD and BMC [34], while one study demonstrated no significant change in either BMD or BMC across the macrocycle [33]. Where site-specific data were reported, no consistent pattern in skeletal adaptation was observed across sport impact categories. Improvements were most frequently noted in leg and arm BMD, while spine, pelvis, and trunk BMD were more commonly unchanged across studies.

Across included studies, the timing and frequency of DXA measurements varied substantially. Seven studies assessed BMD and/or BMC at two timepoints across a macrocycle, typically comparing preseason or off-season measurements with end-of-competition phase assessments. The time between measurements ranged from approximately three to ten months. Several studies employed shorter measurement intervals (≤3–4 months) [30,31,36,39], while others assessed bone outcomes across longer competitive periods (≥6 months). Two studies did not report the time interval between DXA scans [32,34]. Measurement schedules also differed in relation to macrocycle phase, with studies comparing off-season to competition phase, preseason to end-of-competition phase, or multiple in-season timepoints.

Table 3 demonstrates the quality assessment rating for each of the 14 studies included in the review. Twelve studies were rated “good”, and two studies were reported as “fair”-quality. No papers were rated as poor-quality. Full consensus was reached on all ratings with no discrepancies requiring arbitration.

## 4. Discussion

The majority of studies included in this systematic review reported either no significant change or improvements in BMD and/or BMC across the macrocycle in premenopausal female athletes [29,30,31,32,33,37,38,39,40,42], with only one [35] of 14 studies reporting significant BMD declines. Notably, Stanforth et al. [36], Carbuhn et al. [34], and Pettersson et al. [41] each reported both unchanged or improved values within the same cohort depending on sport or athlete subgroup, suggesting that bone responses across the macrocycle are sport-specific rather than uniform. Although seemingly positive results were reported across studies, these findings should be interpreted cautiously given the limited assessment of key confounders and the heterogeneity of study designs. Notably, the absence of change in many studies may reflect insufficient follow-up periods for detecting bone remodelling via DXA [44] or near-maximal bone mass already achieved through years of training [45], rather than definitively indicating preserved bone health. In addition, interpretation is limited by inconsistent reporting of key confounding factors and methodological issues, particularly the inconsistencies in measuring BMD at sport-specific skeletal sites (via DXA) and accounting for training loads, dietary intake, and menstrual cycle status.

### 4.1. High-Impact Sports

Across high-impact sports, studies reported either unchanged or improved BMD and/or BMC. Volleyball and netball athletes predominantly demonstrated improvements in BMD and/or BMC across the macrocycle [29,31,34]. Basketball athletes mostly demonstrated improvements across the macrocycle [29,31], with one study reporting no significant change [27]. Gymnasts similarly reported no significant seasonal changes in BMD [29]. Power athletes, including sprinters and jumpers, demonstrated significant improvements in total BMD, BMD Z-score, and leg BMC [36], consistent with the high-magnitude mechanical loading demands of these sports. The predominantly positive findings are consistent with the established osteogenic effects of jumping, landing, and directional changes [46,47]. However, the absence of change in some athletes likely reflects that highly trained athletes may have already achieved near-maximal BMD [45,48], with maintenance rather than further improvement being the expected adaptive response across a macrocycle.

### 4.2. Odd-Impact Sports

Findings across odd-impact sports were mixed, reflecting the heterogeneous mechanical loading profiles of these sports. Rugby athletes demonstrated divergent results, with Curtis et al. [38] reporting significant increases in BMC with unchanged BMD in rugby sevens athletes, while Yao et al. [37] reported no significant change in BMC throughout a rugby union season. Neither study measured lower body-specific BMD or BMC despite these sports predominantly loading the lower extremities. Soccer athletes consistently reported no significant change in BMD and/or BMC across the macrocycle [31,36]. Softball athletes demonstrated improvements in both BMD and BMC [34], while lacrosse athletes reported significant BMC increases with unchanged BMD [30]. Handball athletes demonstrated significant increases in total body, upper limb, and lower limb BMC across the macrocycle [40]. The absence of lower body-specific measurements in rugby studies and inconsistencies in reporting upper body-specific measurements in throwing sports, further limit understanding of sport-specific skeletal adaptation in these populations.

### 4.3. Repetitive Low-Impact Sports

Repetitive low-impact sports were represented by only two studies in this review, both examining endurance runners, which limits the conclusions that can be drawn for this sport category. The divergent findings may partly reflect differences in competitive level [49,50] and training characteristics [25]. Infantino et al. [35] assessed collegiate athletes across two consecutive competition phases within one year, whereas Pettersson et al. [41] assessed national- and international-level athletes over a single six-month period. The back-to-back competitive season design of Infantino et al. [35] raises questions about the cumulative physiological demands of consecutive competition phases and the ability of bone to recover and adapt between loading blocks [10,11]. These population and methodological differences highlight that endurance athletes cannot be considered a homogeneous group with respect to bone health responses across the macrocycle.

### 4.4. Low-Impact Sports

The largely positive bone health outcomes in rowing and swimming are notable given the non-weight-bearing nature of both sports. In rowing, the divergence between Kurgan et al. [42] and Young et al. [33] may reflect differences in resistance training volume and intensity between competitive levels. Kurgan et al. [42] reported athletes completed an average of 4.5 h of resistance training per week, representing approximately 25% of total weekly training volume, whereas Young et al. [33] reported some training phases only included 1–2 weekly sessions below 60% one-repetition maximum—unlikely to provide sufficient stimulus for further bone adaptation in already-trained athletes. In swimming, both included studies reported improvements in BMD across the macrocycle [34,36], though neither reported training data for their swimming cohorts, limiting mechanistic interpretation of these findings. Notably, Carbuhn et al. [34] reported significant increases across all regional BMD sites despite stable total body BMD, suggesting that total body DXA measures may mask meaningful site-specific adaptations and highlighting the importance of regional bone assessment in repetitive non-impact sporting populations.

### 4.5. Confounding Variables

The substantial underreporting of key confounders across included studies critically limits interpretation of the bone health findings in this review. Only two studies attempted to account for all three primary confounders—menstrual cycle status, dietary intake, and training load—concurrently [30,35], while seven reported no confounding data. As outlined previously, menstrual cycle dysfunction and low energy availability are established mechanistic drivers of bone loss in female athletes [12,17,21], yet limited reporting of menstrual status across included studies prevented determining whether athletes with maintained or improved BMD were predominantly eumenorrheic. Contraceptive use adds further complexity, with evidence remaining equivocal—some research reported improved BMD in female endurance athletes [51] while others found significant decreases in adolescent athletes [52] and amenorrhoeic athletes [53]. Without consistent reporting of menstrual cycle characteristics and contraceptive use, the influence of these factors on seasonal bone health changes cannot be determined.

Low energy availability is perhaps the most critical modifiable factor affecting bone health in female athletes, as it directly suppresses anabolic hormones and amplifies bone resorption independent of menstrual status [21,54]. Despite this, dietary intake was reported in only four studies [30,32,35,42]. The single study demonstrating BMD declines [35] also assessed energy availability, identifying significant associations between EA and BMD in female runners. The failure of the remaining studies to assess dietary intake means it is difficult to determine whether bone health maintenance reflected adequate energy availability or masked underlying energy deficiency.

Training load is a key determinant of skeletal adaptation, yet only three studies attempted to quantify it, each using varied methodologies—including training impulse (TRIMP) [42], validated physical activity questionnaires [35], and accelerometry-based activity monitors [30]. This methodological inconsistency prevents any meaningful comparison of training load across studies or correlation of loading patterns with bone health outcomes. Furthermore, none of the included studies formally examined the relationship between training load and BMD or BMC outcomes through correlation or regression analyses. Critically, of the included studies, training types were not distinguished when quantifying load—a significant limitation given that on-feet weight-bearing activity, off-feet non-weight-bearing activity, and resistance training impose fundamentally different mechanical stimuli on bone [10,11,43]. Session rating of perceived exertion (s-RPE) [55] combined with sport-specific training type classification would provide a more meaningful and practically accessible measure of bone-relevant loading than generic volume or energy expenditure metrics. Without this level of specificity, it remains unclear whether bone health changes across the macrocycle reflect responses to competition demands, resistance training adaptations, or the cumulative effect of uncharacterised loading variation.

### 4.6. Methodological Considerations

Beyond inadequate confounder reporting, several methodological limitations affect interpretation of the findings in this review. First, the timing of DXA measurements varied considerably across studies. Several studies employed measurement intervals as short as three months, which may be insufficient to detect meaningful bone remodelling given that complete remodelling cycles require approximately four to six months [44]. Two studies did not report the time between measurements, further limiting interpretation.

Second, Z-scores were reported in only two studies [25,36]. In athletic populations participating in bone-loading sports, Z-scores above zero would be expected given the osteogenic stimulus of regular training [15]. Athletes presenting with Z-scores in the 0 to −1 range may therefore be classified as having normal bone health when, relative to sport-specific expectations, their values warrant closer clinical attention. The infrequent reporting of Z-scores across included studies limits identification of at-risk individuals and represents a missed opportunity for more clinically meaningful bone health surveillance.

It is important to acknowledge the heterogeneity within this sample: collegiate NCAA athletes and internationally competitive elite athletes differ meaningfully in training exposure, competition frequency, athlete support structures, and dietary monitoring, all of which may influence bone outcomes. These differences are insufficiently captured by the pooled narrative, and findings should not be assumed to generalise uniformly across both populations.

Finally, it is important to acknowledge a limitation of the risk-of-bias tool applied in this review. The NIH Quality Assessment Tool rates overall methodological quality rather than isolating specific domains such as confounder reporting. As a result, studies reporting only a single confounding variable received comparable ratings to those reporting on multiple variables, with no differentiation in quality score based on the comprehensiveness of confounder assessment. Studies may therefore receive comparatively high ratings despite inadequate assessment of key confounding variables, and the quality ratings in Table 3 should be interpreted alongside the confounder reporting data in Table 2 when drawing conclusions about the strength of the evidence base.

### 4.7. Practical Applications

Several practical recommendations emerge from this review. Female athletes in repetitive low-impact sports warrant priority monitoring of energy availability and menstrual cycle regularity given the elevated risk of bone health deterioration in this population. Across all sport categories, practitioners should assess dietary intake and menstrual status alongside DXA outcomes rather than relying on bone density values alone, as stable BMD does not preclude underlying energy deficiency or hormonal dysfunction. Finally, training load monitoring should distinguish between weight-bearing, non-weight-bearing, and resistance training components, as these modalities impose fundamentally different stimuli on bone and generic load metrics are insufficient for informing bone health management in athletic populations.

## 5. Conclusions

Three conclusions from this systematic review are well-supported by the available evidence. First, bone health is predominantly unchanged across the sport macrocycle in highly trained premenopausal female athletes spanning a range of impact sports, with significant BMD declines reported in only one of fourteen studies. Second, the assessment of key confounders—menstrual cycle status, dietary intake, and training load—is critically inadequate across the current literature, with seven studies reporting no confounding data. Third, athletes in repetitive low-impact endurance sports, particularly distance runners, represent a higher-risk subgroup warranting closer monitoring of energy availability and bone health. Several important questions remain unresolved. It is unclear whether the apparent maintenance of BMD reflects true skeletal stability or the insensitivity of DXA over short follow-up periods, near-maximal bone mass in already well-trained athletes, or measurement variability. The degree to which NCAA and internationally competitive elite athletes respond differently across the macrocycle cannot be determined due to insufficient sport- and level-specific reporting. It is clear, however, that the field cannot reliably identify athletes at risk without systematic reporting of menstrual cycle status, energy availability, and training load alongside sport-specific DXA protocols. Until these variables are treated as methodological requirements rather than optional additions, conclusions about seasonal bone health in female athletes will remain limited in their translational value for practitioners and clinicians working in high-performance sport.

## Figures and Tables

**Table 1:** PICO framework.

1. Population	Premenopausal, highly trained female athletes, encompassing both elite (nationally and internationally competitive) and collegiate (NCAA) populations Participating in competitive sports (both impact and non-impact sports)
2. Exposure	Participation in competitive season training and competition Exposure to seasonal training loads, competition phases, and associated physiological demands
3. Comparison	Temporal comparison: longitudinal within-athlete measurements across one or more macrocycle phases, including offseason, preseason, in-season, and post-season timepoints Within-subject changes over time
4. Outcome	Primary: Bone mineral density (BMD) measured by dual-energy X-ray absorptiometry (DXA) Secondary: Bone mineral content (BMC)

**Table 2:** Overview of study findings assessing bone changes in female athletes across respective sporting macrocycles.

	Population	Sport/s Assessed	Skeletal Site	Macrocycle Phase of DXA Scans	Time Between DXA Scans	Results	Confounding Variables Included	Change	
								BMC	BMD
High-Impact Sports									
Moore et al., 2024 [29]	Female (*n* = 20) athletes aged 19.1 ± 1.2 years.	NCAA Division I gymnasts	- TB BMD	Start of competition phase End of competition phase	6 months (approx.)	Unchanged BMD across phases (*p* = 0.869).	Menstrual cycle	n/a	↔
Pettersson et al., 2024 [41]	Female (*n* = 15) athletes aged 22.2 ± 2.8 years.	Swedish national and international power athletes (sprinters and jumpers; *n* = 32)	TB BMD BMC - TB - Arm - Trunk - Leg - Z-scores	Off- season phase Mid-competition phase	6 months (approx.)	Significant improvements in TB BMD, BMD Z-score, TB BMC, and legs BMC between P1 and P2 (*p* ≤ 0.03) Unchanged arm and trunk BMC (*p* < 0.05)	x	↑/↔	↑
Hogarth et al., 2021 [39]	Female (*n* = 20) athletes aged 26.5 ± 4.7 years.	Elite netball players (Suncorp Super Netball)	- TB BMD - TB BMC	Start of Off-season phase Middle of preseason phase End of preseason phase Post-competition phase.	3 months (approx.)	TB BMD significant improvement between P2 and P4 (*p* = 0.02) TB BMC significant improvement between P1 and P3 (*p* = 0.01)	x	↑	↑
Stanforth et al., 2016 [36]	Female athletes (*n* = 113) aged 20.1 ± 0.1 (SE)	NCAA Division 1 basketball (BB) (*n* = 38); volleyball (VB) (*n* = 26) athletes; track and field (TF) (*n* = 49) athletes	BMD - TB - Spine - Pelvis - Trunk - Arm - Leg - Spine- - Pelvis - Trunk - Arm - Leg	Start of preseason phase End of competition phase	BB: 5–6 months VB: 3–4 months	BB: Significant improvement in TB BMC and, TB, arm and leg BMD from P1 to P2 (*p* < 0.05) Unchanged spine, pelvis and trunk BMD (*p* > 0.05) VB: Significant improvement in TB, arm, pelvis and leg BMD from P1 to P2 (*p* < 0.05) Unchanged spine and trunk BMD (*p* > 0.05) TF: Significant improvements in leg, pelvis and spine BMD across phases (*p* < 0.05)	x	↑	↑/↔
Nepocatych et al., 2017 [32]	Female athletes (*n* = 10) aged 20 ± 1 years.	NCAA Division I basketball athletes	BMD - TB - Spine - Femur	Start of competition phase End of competition phase	Not reported	Unchanged BMD between phases (*p* > 0.17)	Dietary intake	↔	n/a
Carbuhn et al., 2010 [34]	Female athletes (*n* = 34) aged 20 ± 2 years	NCAA Division 1, basketball (*n* = 10)l, volleyball (*n* = 7), track jumpers and sprinters (*n* = 17)	TB BMC BMD - TB - Arm - Leg - Pelvis - Spine	Off-season phase Preseason phase Post competition phase	Not reported	BB: significant increase in BMD between P1 and P3 (*p* = 0.05) Significant reduction in spine BMD between P1 and P2 (*p* < 0.05) Unchanged BMC across all phases (*p* < 0.05) VB: significant increase in TB BMC, TB BMD, leg BMD between P1 and P3 (*p* < 0.05) Significant increase in spine BMD (P2–P3) (*p* < 0.05) Arm and pelvis BMD unchanged (*p* > 0.05) TF: significant increases in all measures from P1 to P3 (*p* < 0.05) Significant improvement in leg and pelvis BMD (P1–P2 and P2–P3) (*p* < 0.05) All other variables unchanged between P1 and P2 and P2 and 3 (*p* > 0.05)	x	↑/↔	↑/↔
Odd-Impact Sports									
Yao et al., 2024 [37]	Female (*n* = 42) athletes aged 28 ± 6 years.	Elite rugby union players (English Premiership)	- TB BMC	Preseason phase Mid-competition phase End of competition phase	4–5 months (approx.)	Unchanged BMC across all phases (ES = −0.02–0.14)	x	↔	n/a
Curtis et al., 2021 [38]	Female athletes (*n* = 15) aged 27 ± 5 years.	Elite rugby 7s players (Premiership League)	- TB BMD - TB BMC	Start of competition phase End of competition phase competitive season.	7 months	Unchanged TB BMD between P1 and P2 (*p* > 0.05) Significant improvements in TB BMC between P1 and P2 (*p* < 0.05)	x	↑	↔
Stanforth et al., 2016 [36]	Female athletes (*n* = 47) aged 19.7 ± 0.1 (SE)	NCAA Division 1 soccer athletes	TB BMC BMD - TB - Spine - Pelvis - Trunk - Arm - Leg	Start of preseason phase End of competition phase	2–3 months (approx.)	Unchanged BMD/BMC between P1 and P2 (*p* > 0.05)	x	↔	↔
Zabriskie et al., 2019 [30]	Female (*n* = 20) athletes aged 20.4 ± 1.8 years	NCAA Division II lacrosse players	- TB BMD - TB BMC - Z-score	Start of offseason phase Start of preseason phase End of competition phase	4–5 months (between P1 and P2) 3–4 months (between P2 and P3)	Significant improvement in BMC from P1 to P3 and P2 to P3 (*p* < 0.001) Significant improvement in z-score from P1 to P3; P2 to P3 (*p* = 0.04) Unchanged TB BMD across phases (*p* = 0.167)	Menstrual cycle Dietary intake Physical activity monitoring	↑	↔
Minett et al., 2017 [31]	Female (*n* = 24) athletes aged 19.0 ± 0.2 years.	NCAA Division I soccer players	- Hip BMD - Femoral neck BMD	End of preseason phase End of competition phase End of offseason phase	3 months (approx. between P1 and P2) 6 months (approx. between P2 and P3)	Unchanged BMD between phases (p value not reported)	Menstrual cycle	n/a	↔
Nepocatych et al., 2017 [32]	Female athletes (n= 20) aged 20 ± 1 years.	NCAA Division I softballers	BMD - TB - Spine - Femur	Start of competition phase End of competition phase	Not reported	Unchanged BMD between phases (*p* > 0.17)	Dietary intake	n/a	↔
Milanese et al., 2012 [40]	Female (*n* = 43) athletes aged 22.8 ± 6.49.	Italian national handball team (elite level, *n* = 26 and sub-elite level, *n* = 17).	BMC - TB - Upper body - Trunk - Lower body	Start of competition phase End of competition	8 months (approx.)	Significant increases in TB BMC, upper body BMC, and lower body BMC from P1 to P2 (*p* < 0.0001)	x	↑	n/a
Carbuhn et al., 2010 [34]	Female athletes (*n* = 17) aged 20 ± 1	NCAA Division 1 softball athletes	TB BMC BMD - TB - Arm - Leg - Pelvis - Spine	Off-season phase Preseason phase End of competition phase	Not reported	Significant increases in BMD TB between P1 and P2 (*p* < 0.05) Significant increase in BMC between P1 and P3 (*p* < 0.05) Significant increase in spine BMD between P1 and P3 (*p* < 0.05) All other site-specific BMD unchanged across phases (*p* > 0.05)	x	↑	↑/↔
Repetitive Low-Impact Sports									
Pettersson et al., 2024 [41]	Female (*n* = 12) athletes aged 23 ± 3.8 years.	Swedish national and international level athletes from endurance (800–10,000 m)	TB BMD z-scores BMC - TB - Arm - Trunk B - Leg	Off-season phase Mid-competition phase	6 months (approx.)	Unchanged total BMC and BMD (*p* > 0.05) Significant improvements in BMD Z-score and legs BMC between P1 and P2 (*p* ≤ 0.03)	x	↔	↔
Infantio et al., 2021 [35]	Female (*n* = 18) aged 19.2 ± 1.2	NCAA Division I cross-country runners	BMD - TB - Posterior–anterior (PA) spine - Lateral spine - Femoral neck - Total hip	Start of preseason (cross-country) End of competition phase (cross country) End of competition phase (track).	4–6 months (approx. between P1 and P2) 12 months (between P1 and P3)	Significant BMD reduction from P1 to P2 (*p* < 0.047) PA spine Significant BMD reduction from P1 to P3 (*p* = 0.014) Total hip Significant reduction in all BMD from P1 to P3 (*p* < 0.002)	Menstrual cycle Dietary intake Physical Activity questionnaire	↓	n/a
Repetitive Non-Impact Sports									
Kurgan et al., 2018 [42]	Female (*n* = 15) athletes aged 27.0 ± 0.8 years.	Olympic heavyweight rowers	BMD and BMC - TB - Lumbar spine - Rib - Pelvis - Arm - Leg	Start of competition phase End of competition phase	10 months (approx.)	Significant improvement in TB BMD between P1 and P2 (*p* < 0.05) Unchanged site-specific BMD (*p* > 0.06) Unchanged BMC across phases (*p* = 0.34)	Dietary intake Training Load	↔	↑
Stanforth et al., 2016 [36]	Female athletes (*n* = 52) aged 20.1 ± 0.1	NCAA Division 1 Swimming athletes	TB BMC BMD - TB - Spine - Pelvis - Trunk - Arm - Leg	Start of preseason phase End of competition phase	5–6 months (approx.)	Significant improvement in total and leg BMD between P1 and P2 (*p* = 0.05) Unchanged BMC values across phases (*p* > 0.05)	x	↔	↑
Young et al., 2014 [33]	Female (*n* = 5) aged 22.4 ± 5.5	NCAA Division 1 heavyweight rowers	BMD and BMC - Lumbar spine	Start of competition phase Middle of competition phase End of competition phase.	9 months (approx.)	Unchanged BMD and BMC across phases (ES = 0.05)	Menstrual cycle	↔	↔
Carbuhn et al., 2010 [34]	Female athletes (*n* = 16) aged 19 ± 1 years	NCAA Division 1 swimming athletes	TB BMC BMD - TB - Arm - Leg - Pelvis - Spine	Preseason phase End of competition phase	Not reported	Significant increase between P1 and P2 across all measures (*p* < 0.05) Unchanged TB BMD across phases (*p* > 0.05)	x	↑	↑

Legend: x = study did not report on confounding factor; ↔ no significant change in BMD/BMC value between timepoints; ↑ significant increase in BMD/BMC value between timepoints; ↑↔ mixed results reported between total and site-specific values; ↓ significant decrease in BMD/BMC value between timepoints; n/a = not assessed. TB = total body; BB = basketball; VB = volleyball; TF = track and field.

**Table 3:** Studies included in the Quality Assessment Tool.

Author, Year	Research Question	Study Population	Participation Rate ≥ 50%	Inclusion Criteria	Sample Size Justification	Expose Before Outcome	Timeframe Appropriate	Exposure Examined	Exposure Measures Valid/Reliable	Exposure Assessed More Than Once	Outcome Measures Valid/Reliable	Loss to Follow-Up ≤ 20%	Confounders Measured/Adjusted	Overall Rating
Carbuhn et al., 2010 [34]	✔	✔	✔	✔	x	✔	✔	✔	✔	✔	✔	✔	x	Good
Curtis et al., 2022 [38]	✔	✔	✔	✔	x	✔	✔	x	✔	✔	✔	✔	x	Fair
Hogarth et al., 2021 [39]	✔	✔	✔	✔	x	✔	✔	✔	✔	✔	✔	✔	x	Good
Infantino et al., 2021 [35]	✔	✔	✔	✔	x	✔	✔	✔	✔	x	✔	✔	✔	Good
Kurgan et al., 2018 [42]	✔	✔	✔	✔	✔	✔	✔	✔	✔	x	✔	✔	✔	Good
Milanese et al., 2012 [40]	✔	✔	✔	✔	x	✔	✔	✔	✔	✔	✔	x	x	Fair
Minett et al., 2017 [31]	✔	✔	✔	✔	✔	✔	✔	✔	✔	x	✔	✔	✔	Good
Moore et al., 2024 [29]	✔	✔	✔	✔	✔	✔	✔	✔	✔	x	✔	✔	✔	Good
Nepocaytch et al., 2017 [32]	✔	✔	✔	✔	✔	✔	✔	✔	✔	x	✔	✔	✔	Good
Pettersen et al., 2024 [41]	✔	✔	✔	✔	✔	✔	✔	✔	✔	✔	✔	✔	✔	Good
Stanforth et al., 2014 [36]	✔	✔	✔	✔	✔	✔	✔	✔	✔	✔	✔	✔	x	Good
Yao et al., 2024 [37]	✔	✔	✔	✔	x	✔	✔	✔	✔	✔	✔	✔	x	Good
Young et al., 2014 [33]	✔	✔	✔	✔	x	✔	✔	✔	✔	✔	✔	✔	x	Good
Zabriskie et al., 2019 [30]	✔	✔	✔	✔	x	✔	✔	✔	✔	✔	✔	✔	x	Good

## Data Availability

No new data was created in this review; however, Supplementary Material S1 outlines search strategies.

## Supplementary Materials

The following supporting information can be downloaded at: https://www.mdpi.com/article/10.3390/sports14040162/s1, Supplementary S1. Full database search strategies; Supplementary S2. PRISMA checklist.
